# The effects of normovolemic anemia and blood transfusion on cerebral microcirculation after severe head injury

**DOI:** 10.1186/s40635-018-0210-5

**Published:** 2018-11-08

**Authors:** Judith Bellapart, Kylie Cuthbertson, Kimble Dunster, Sara Diab, David G. Platts, Christopher Raffel, Levon Gabrielian, Adrian Barnett, Jennifer Paratz, Rob Boots, John F. Fraser

**Affiliations:** 1 Critical Care Research Group, University of Queensland, Brisbane, Queensland Australia; 2 Intensive Care Department, Royal Brisbane and Women’s Hospital, Butterfield Street, Herston, QLD 4025 Australia; 3 School of Medicine, University of Queensland, Brisbane, Queensland 4025 Australia; 4 Griffith University, Parkland Drive, Southport, 4215 Australia; 5 Histopathology Department, Royal Brisbane and Women’s Hospital, Herston, QLD 4025 Australia; 6 Medical Engineering Research Facility, Queensland University of Technology, Stafford Heights, QLD 4053 Australia; 7 Department of Cardiology, The Prince Charles Hospital, Chermside, QLD 4032 Australia; 8 Medical School Research Centre, Frome road, Adelaide, SA 5005 Australia; 9 Institute of Health and Biomedical Innovation and School of Public Health and Social Work, Queensland University of Technology, 60 Musk Avenue, Kelvin Grove, QLD 4059 Australia; 10 Intensive Care Department, The Prince Charles Hospital, Rode road, Chermside, QLD 4032 Australia

**Keywords:** Anemia, APP staining, Histology, Microcirculation, Microspheres

## Abstract

**Background:**

Cerebral regional microcirculation is altered following severe head injury. In addition to tissue disruption, partial pressure of tissue oxygenation is impaired due to an increase in the oxygen tissue gradient. The heterogenic distribution of cerebral microcirculation is multifactorial, and acute anemia challenges further the delivery of oxygen to tissues. Currently, a restrictive transfusion threshold is globally applied; however, it is unclear how anemia modifies regional cerebral microcirculation; hence, it is unclear if by aiming to a global endpoint, specific anatomical regions undergo ischemia. This study aims to quantify the temporal changes in cerebral microcirculation after severe head injury, under the effect of anemia and transfusion. It also aims to assess its effects specifically at the ischemic penumbra compared to contralateral regions and its interactions with axonal integrity in real time. Twelve ovine models were subjected to a severe contusion and acceleration-deceleration injury. Normovolemic anemia to a restrictive threshold was maintained after injury, followed by autologous transfusion. Direct quantification of cerebral microcirculation used cytometric count of color-coded microspheres. Axonal injury was assessed using amyloid precursor protein staining.

**Results:**

A mixed-effect regression model from pre-transfusion to post-transfusion times with a random intercept for each sheep was used. Cerebral microcirculation amongst subjects with normal intracranial pressure was maintained from baseline and increased further after transfusion. Subjects with high intracranial pressure had a consistent reduction of their microcirculation to ischemic thresholds (20–30 ml/100 g/min) without an improvement after transfusion. Cerebral PtiO_2_ was reduced when exposed to anemia but increased in a 9.6-fold with transfusion 95% CI 5.6 to 13.6 (*p* value < 0.001).

**Conclusions:**

After severe head injury, the exposure to normovolemic anemia to a restrictive transfusion threshold, leads to a consistent reduction on cerebral microcirculation below ischemic thresholds, independent of cerebral perfusion pressure. Amongst subjects with raised intracranial pressure, microcirculation does not improve after transfusion. Cerebral oxymetry is impaired during anemia with a statistically significant increase after transfusion. Current transfusion practices in neurocritical care are based on a rigid hemoglobin threshold, a view that excludes cerebral metabolic demands and specific needs. An RCT exploring these concepts is warranted.

**Electronic supplementary material:**

The online version of this article (10.1186/s40635-018-0210-5) contains supplementary material, which is available to authorized users.

## Background

Severe head injury often combines the effects of a contusion with an acceleration-deceleration force leading to axonal tearing, cytogenic, vasogenic edema, impaired cerebral autoregulation, and subsequent perfusion mismatch [1]. Secondary patho-physiologic processes may ultimately result in areas of ischemia and cerebral infarct. These changes are commonly found in regions where cerebral microcirculation (RMBF) has been critically reduced [2, 3]. An additional contributor to cerebral ischemia is partial pressure of tissue oxygenation (PtiO_2_) with a series of studies focussing on the relevance of cerebral oximetry [4–7]. While current evidence-based practice attempts to minimise the use of allogenic blood transfusions, specifically amongst critically ill patients [8, 9] by establishing a restrictive transfusion threshold [10–13]; controversy still remains regarding the safety of these measures, specifically when facing sustained low PtiO_2_ levels, during the acute phase of head injury [4, 7, 14–19]. Studies have showed that a sustained hemoglobin concentration of 7 g/dL (known as the “restrictive transfusion threshold”) correlates with neurological injury due to cerebral tissue hypoxia [16]. In addition, blood transfusion has shown to significantly increase PtiO_2_ independently of cerebral perfusion pressure [17, 18]. Furthermore, questions continue to rise despite a recent randomised control study [20] not really challenging the proposed level of acceptable anemia at a hemoglobin of 7 g/dL. In the meantime, the absence of clinical equipoise as well as the lack of methodological feasibility in designing a study that focuses on cerebral RMBF during anemia and severe head injury, leads to the need of experimental models, to further challenge this question. In the absence of clinically available measures of cerebral microcirculation, physiological markers of cerebral ischemia and hypoperfusion rather than global metrics such as mortality outcomes may be more appropriate endpoints [21].

This study challenges the following hypothesis:

- Hypothesis one: RMBF after severe head injury is reduced over time, specifically, at the contusion regions when compared to contralateral regions.
- Hypothesis two: The maintenance of normovolemic anemia followed by self-transfusion, does not significantly impair RMBF.
- Hypothesis three: RMBF is most reduced in regions of severe tissue disruption, shown by a high amyloid precursor protein (APP) staining score.
- Hypothesis four: The maintenance of normovolemic anemia significantly reduces cerebral Pti02, these changes revert following self-transfusion.

The aims of this study are described as follows:

- First aim: To quantify the temporal changes in cerebral RMBF after severe head injury in an ovine model, specifically at the ischemic penumbra compared to contralateral regions, using cytometric counting of color-coded microspheres.
- Second aim: To simultaneously superimpose cerebral blood flow data with histopathology data, by using APP staining at each region of interest and at each time point before and after severe head injury.
- Third aim: To quantify cerebral Pti02 through all time-points to demonstrate its changes to anemia and transfusion.

## Methods

### Animal care and preparation

Sheep were the most appropriate animal model, due to their physiological similarities to humans; certain anatomical aspects such as the cerebral gyrencephalic surface of their brains, that allow better examination of the grey-white matter and physiological similarities within the hemoglobin dissociation curve [22]. Additionally, a robust experience using this animal model [23–25] supported its selection. A convenience sample of 12 *Ovis Aries* wethers weighing 40 ± 5 k were instrumented with a triple lumen central line (Cook Medical INC. QLD, Australia) and two 16 Fr introducer sheaths in the right internal jugular (RIJ) vein. General anesthesia was given through the central line: ketamine with an initial bolus of 5 mg/k and maintenance infusion at 0.5–1 mg/k/h; and infusions of midazolam (0.5 mg/k/h), fentanyl (10 mcg/k/h), and alfaxalon (6 mg/k/h), previously used [26] demonstrating that this anesthetic combination maintains cardiovascular stability without altering cerebral microcirculation [27]. Hydration was ensured with Hartmann’s solution, titrated to maintain a central venous pressure (CVP) of 6–10 mmHg. Cardiovascular monitoring included cardiac output and vascular resistances via a Swan-Ganz catheter as previously described [26, 28]. A 5F umbilical vessel catheter (Argyle, Tyco HealthCare, Mansfield, MA, USA), placed in the right femoral artery was used to allow the withdrawal of blood for the referral sample, at a rate of 10 ml/min. Orotracheal intubation used a size 10 mm endotracheal tube (SIMS Portex, UK). Sheep were ventilated at 12 bpm with tidal volumes of 8 mL/kg and 5 cmH_2_O of PEEP with an initial FiO2 of 1.0. FiO2, and respiratory rate were titrated to maintain a partial pressure of oxygen (PaO_2_) of > 95 mmHg and normocapnia. PEEP levels were maintained at 5 cmH_2_O to minimise de-recruitment while known not to effect on cerebral blood flow [26, 29].

Neuro-monitoring used a Lycox PtiO_2_ probe and an intracranial pressure (ICP) monitor, Oxford Optronics, Ltd., Oxford, UK. Craniectomies were performed prior to injury but dura was not pierced until after the injury was elicited, to avoid the potential vent of intracranial pressure. Craniotomies for the insertion of both probes were performed exactly 15 mm lateral to the sagittal suture and anterior to the coronal suture [25, 30]. Probes were introduced at 35 mm and 15 mm from the skull respectively with the end of the tip located at the white matter as previously performed [31].

To avoid red blood cell storage into the sheep’s spleen and maintaining stable hemoglobin throughout the study [32, 33], the ligation of the splenic artery was performed as reproduced from previous studies [26, 31].

The monitoring and preparation phase was completed with an intracardiac echocardiography (ICE) guided insertion of a transseptal catheter into the left cardiac atrium (LA). Echocardiography images were obtained using an Acuson Sequoia C512 scanner (Siemens, California). Transeptal puncture and insertion of a pigtail catheter into the LA followed previously described methods [34].

### Trauma model

Under non-recovered anesthesia and with the animal’s head in a sphinx’s position, a blunt injury was applied over the left temporal bone using a non-penetrating stunner (model MKL, Karl Schermer, Ettlingen Germany) with the intention to induce a severe head injury. This model followed the same methodology than in a previous study [26], leading to a final combination of contusion and acceleration injury [35].

### Normovolemic anemia model

The targeted hemoglobin goal was defined at each subject, depending upon their post-spleen ligation hemoglobin levels. The aim was to achieve a 30% reduction on the animal’s hemoglobin, from their baseline. Baseline hemoglobin was defined as the post-spleen ligation hemoglobin once stability of the intravascular hemoglobin was achieved. The rationale behind the achievement and maintenance of such reduction was to reproduce the same proportional decay in hemoglobin as the “anemia threshold” previously described in the TRICC Trial [10]. Acute normovolemic anemia was achieved by sequential blood extractions from the indwelling arterial catheter, with simultaneous isovolemic saline infusions to ensure normovolemia. Extracted blood was stored into a Leukotrap ® RC System (Baintree MA, 02184, USA) and maintained at room temperature. The cardiovascular response to anemia was monitored using cardiac index, blood pressure and systemic vascular resistances to ensure that the shift on intravascular volume was not leading to intravascular depletion. Regular arterial blood gas sampling was used to ensure stable and normal lactate levels; cardiac monitoring was recorded continuously and in real time while blood gases were performed every hour.

### Self-transfusion model

Once the targeted anemia had been achieved and maintained for over 2 h allowing physiological and metabolic responses to establish, animals were self-transfused. Labelled and identified Leukotrap ® bags were selected for each subject and connected to their central lines. Blood was transfused over 30 min while cardiovascular monitoring was continuously recording. An arterial blood gas was performed before, during and after the transfusion to avoid over-transfusion, specifically if hemoconcentration was present, in which case, 250 ml volume crystalloid aliquots was administered to achieve the level of hemoglobin closest to each baseline value.

### Protocol for microspheres injection

Interventions were distributed along different time epochs, as shown in Table 1. Along these time points (T0–T4) an injection of color-coded microspheres (E-Z TRAC; Interactive Medical Technology, Los Angeles, CA, USA) was done through the LA pigtail catheter as previously performed [26, 31, 36]. Randomly assigned color at each time-point and subject minimised selection biases and allowed the tracking of RMBF at specific anatomical regions for each time-point and subject. Five different colors (purple low, purple high, pink high, yellow high, and coral low) were recommended by the manufacturer www.microspheres.net to facilitate cytometric count. Each injection included a homogeneous mixture of one color-microspheres of a density equal to 5 million spheres in a 0.8 mL. This microsphere density has been seen not to cause microvascular occlusion [37].

**Table 1:** Final study (4 and 5 of 5 studies) interventions distributed along different time points

Time	Series of chronological interventions
	Central lines, intubation, and ventilation set up.
	Infusions and intracardiac catheterization.
	Arterial catheter and withdrawal pump set up.
	Spleen ligation.
T0	*Baseline* injection of color-coded microspheres
	Burr-holes completion
	*Head injury model*
	PTi02 and ICP proves insertion
T1	First injection of color-coded microspheres
	*Anemia model*
T2	Second injection of color-coded microspheres
T3	Third injection of color-coded microspheres
	*Self-transfusion model*
T4	Fourth injection of color-coded microspheres
	*Euthanasia and organ retrieval*

Italics refer to the phases through which each experiment goes through

Microspheres were injected 30 s after the initiation of the withdrawal pump. The withdrawal pump was connected to the arterial catheter with the intention to withdraw blood at a rate of 10 mL/min to obtain the reference blood sample required for the calculation of regional tissue RMBF. 2 min after commencement of the withdrawal pump the reference blood sample collection was completed, and the inline catheter was flushed with Tween 80 reagent to recover all the microspheres that could be entrapped in the line [38].

### Euthanasia and post-mortem tissue manipulation

At the end of the study time and approximately 30 min after T4 allowing a systemic distribution of microspheres, sheep were euthanized under non-recovered anesthesia with a bolus injection of 0.5 mL/k of sodium pentobarbitone. After confirmation of death (defined as asystole), the brain was extracted, weighed, and fixed with 10% formalin for 3 weeks.

### Brain harvesting technique

Brain harvesting required the use of a round reciprocating saw to facilitate skull sections of approximately 5 cm. These sections were performed from the frontal region to the posterior fossa. These bone sections were removed whilst simultaneous dissection of the dura avoided parenchymal tearing. Once the brain was fully exposed, the olfactory bulbs, optic chiasm, tentorium, and cranial nerves were progressively sectioned as the brain was lifted from the base of the skull. This approach achieved a controlled dissection avoiding injury to the brain tissue and ensuring a cautious brain removal as previously demonstrated [27]. The brains were weighed prior to insertion into a formalin bath for an immersion fixation during a minimum of 3 weeks.

### Tissue sampling model

After 3 weeks minimum of immersion fixation, the brains were macroscopically inspected to assess for cortical impacts, hemorrhages or the presence of contra-coup injury. Following external inspection, each brain was sectioned creating 5 mm antero-posterior slices. Each slice was macroscopically inspected to identify regions of maximal contusion. Cone samples were extracted from pre-defined anatomical regions (Table 2) as previously published [26, 31]. Adjacent tissue blocks were assigned for both cytometric and histological analysis, to superimpose histology with cerebral blood flow data and allow a flow/tissue cerebral mapping.

**Table 2:** Tissue sampling labelling

Anatomical regions	Anatomical location
AL	Core of contusion, left side
BL	Ischemic penumbra, left side
AR	Mirror region to core of contusion, on the right
BR	Mirror region to ischemic penumbra, on the right
C	Thalamus ipsilateral to injury
D	Medulla

Samples from the skin, kidney, heart, and spleen were extracted from each sheep to demonstrate systemic distribution of microspheres as well as to confirm the presence of splenic infarct related to a successful spleen ligation respectively.

### Quantification of microvascular blood flow

Cytometric analysis directly quantified the amount of microspheres of each particular color at each specific region of interest [39]. RMBF was calculated as a mathematical derivation from the microsphere concentration that was injected into the heart at each injection time and the amount of each color microspheres found at each “reference sample of blood.” The reference sample of the blood is the arterial blood sample withdrawn at a known rate over a fixed period of time. RMBF represents the proportion of microspheres trapped in the targeted tissue in relation to the total quantity of spheres per milliliter of blood per minute on the reference sample, it uses the following equation:

RMBF (mL/min/g) = (total tissue spheres)/[(tissue weight, g) × (reference spheres/mL/min)] [40].

Cytometric analysis was performed at the Interactive Medical Technology (IMT), Los Angeles, CA, USA, www.microspheres.net.

### Immunohistochemistry processing

Immunohistochemistry analysis was performed at the neuro-pathology laboratory, Royal Brisbane and Women’s’ Hospital, QLD, Australia. Immunohistochemistry used a Leica Novolink Polymer Detection Systems Kit (Leica Microsystems Pty Ltd., North Ryde, 2113 Australia) as per manufacturer’s instructions, www.leica-microsystems.com. Sections had paraffin removed through a series of xylene immersions and re-hydrations. Antigen retrieval was carried out using Leica BOND ER1 solution. Sections were incubated with a protein block. The primary antiserum made up in Leica BOND Antibody Diluent was applied to the sections.

### Immunohistochemistry and hematoxillin and eosin scoring, and interpretation

Immunohistochemistry analysis using APP antibodies staining was applied to all targeted areas of interest. APP antibody staining was used to identify areas of tissue with high density of APP staining, specifically at regions of interest. APP expression is considered to be a very early marker of neuronal damage [41] and therefore suitable as an early histopathological marker, interesting in the setting of this 4-h study. A grading system of the density of APP staining [27] was used. APP score was structured into three qualitative categories dependent upon the severity of injury seen: *mild*, a focal contusion with APP labelling limited to the site of injury or focal APP labelling; *moderate*, a pattern of APP staining greater than one hemisphere, greater than half a hemisphere or less than half a hemisphere; *severe*, characterised for the presence of diffuse staining and sub-classified as either diffuse vascular injury, diffuse axonal injury with macroscopic hemorrhage, diffuse axonal injury with microscopic hemorrhage/tissue tears or diffuse axonal injury only [27]. Each animal had samples for both cytometric count of RMBF and immunohistochemistry at each anatomical region of interest with the intention to superimpose flow data with histopathology data at each area of interest and at each time point before and after severe head injury.

### Statistical analysis

RMBF raw data for each sheep was plotted over time and then time averaged for the study cohort. The ratio of RMBF from 1 to 4 h after injury (T1–T4) compared to baseline (time zero–T0) was plotted. A ratio of one indicated no RMBF changes pre-post injury. RMBF values below the ratio of one indicted that RMBF was reduced over time from baseline; RMBF values above the ratio indicated an increase in RMBF over time from baseline. To test for statistical differences, we used a mixed-effect regression model of the ratios from times T1 to T4 with a random intercept for each sheep to control for repeated responses from the same sheep. We fitted an independent effect at each time (T1 to T4) as we were uncertain of how the change in RMBF over time would be. We also examined a simpler model where the RMBF ratio was the same at times T1 to T4.

All the plots and regression models were run separately for each area studied (AR, BR, AL, BL, C and D). We used the R software version 3.1.2 for all analyses.

Statistical analyses also assessed the differences between RMBF after head injury during the anemia phase (T2 and T3) and after blood transfusion (T4).

## Results

**Fig. 1:**
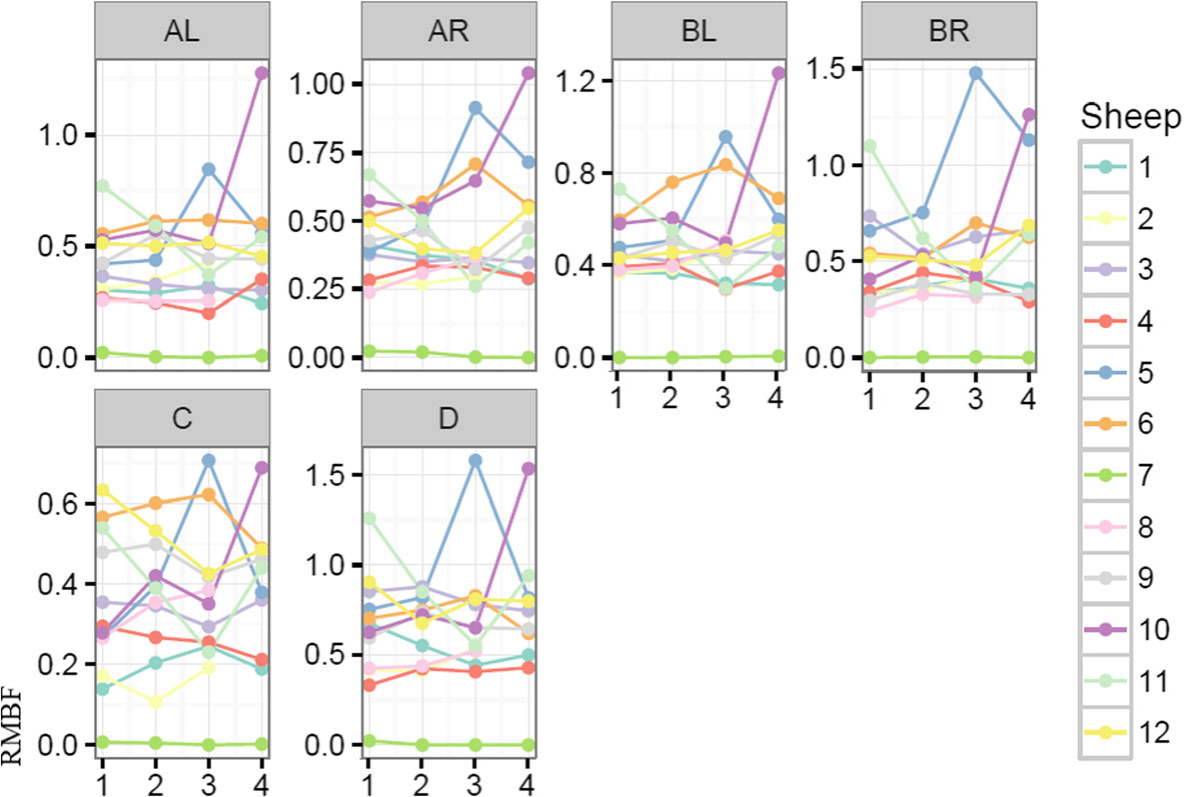
Regional microcirculation blood flow (RMBF) ratio from baseline for all subjects at each anatomical region of interest over time. Time-points (T1–T4) on the *x* axis; RMBF per 1 g tissue on the *y* axis

A convenience sample of 12 subjects weighing 40–45 k was used in this study. All subjects except one (subject number 7) remained cardio-vascularly stable throughout the study time (Table 3). Subject number 7 became hypotensive requiring metaraminol infusion to support his cerebral perfusion pressure (CPP); such profound vasoplegia immediately after trauma was suggestive of cerebral herniation. This observation is corroborated by the cytometric quantification of RMBF that demonstrated undetectable RMBF at all time-points and regions during the study (Fig. 1). Systemic variables affecting CPP and reflecting peripheral oxygen extraction, such as cardiac output (CO) and central venous oxygen saturation (SVc02) respectively, were maintained homogeneously within each subject for the entire study period (Table 3). A specific attention was given to normocapnia with the goal of avoiding hypocapnia due to its known effects over cerebral blood vessel calibre, leading to vasoconstriction with the potential contributory effect of hypoperfusion. Metabolic variables reflecting oxygen delivery to tissues as well as impacting on microcirculation rheology, such as the hemoglobin level is represented in Table 4 (Additional file 1) . During T2 and T3, a 30% reduction in each subject’s baseline hemoglobin was achieved as per protocol. Subsequent self-transfusion was completed on T4, showing the increment on hemoglobin in 8 of the 12 subjects studied. PtiO_2_ was recorded in every subject from T1, as PtiO_2_ probes were inserted after craniectomies were formalised (Table 5). PtiO_2_ at T1 was seen to very critically low in some subjects; this is a common observation following the insertion of the probe. In seven of 12 subjects, PtiO_2_ values were persistently below the described ischemic threshold [4–7] throughout the trauma and anaemia phase. In all the subjects except for two (subject numbers 2 and 7), PtiO_2_ values significantly incremented and recovered after self-transfusion. At time T4, the PtiO_2_ was 9.6 times higher on average, 95% CI 5.6 to 13.6 (*p* value < 0.001), this observation is consistent with previous studies.

**Table 3:** Mean arterial blood pressure (mmHg), intracranial pressure and cerebral perfusion pressure (mmHg) mean values in all subjects at each time point

MAP/ICP/CPP (mmHg)	T0 pre-injury	T1	T2	T3	T4
SV02 (%)/CCO (L/min)	MAP				
**Subject 1**	97	114/**43**/71	76/**23**/53	74/**24**/50	79/**29**/50
	72/3.1	75/3.0	74/2.6	77/3.6	84/*4.7*
**Subject 2**	98	74/**23**/51	91/**30**/61	79/**29**/50	92/**42**/50
	NA/2.5	NA/2.4	NA/2.5	NA/2.5	NA/*3.0*
Subject 3	110	100/NA/NA	90/NA/NA	130/NA/NA	125/NA/NA
	75/3.4	79/3.4	66/2.9	69/3.1	77/*4.1*
**Subject 4**	117	106/**23**/83	137/**23**/114	130/**23**/107	135/**25**/110
	70/4.6	70/4.7	71/3.8	72/3.9	74/*5.7*
**Subject 5**	114	97/**23**/74	72/**21**/51	79/**25**/54	94/**27**/67
	85/4.0	79/3.6	78/3.7	80/3.7	86/*5.9*
Subject 6	90	99/12/87	100/13/87	92/13/79	81/10/71
	77/3.2	73/3.2	66/2.3	61/2.3	86/*5.7*
**Subject 7**	90	70/**22**/48^a^	70/**35**/35^a^	80/**46**/34^a^	90/**67**/23^a^
	92/4.6	98/4.1	98/4.7	97/3.3	98/3.9
Subject 8	110	80/7/73	79/6/73	90/4/86	95/4/91
	83/4.8	87/4.3	87/5.1	86/4.5	87/*6.2*
Subject 9	90	90/8/82	94/6/88	95/5/90	103/4/99
	54/2.9	51/3.1	53/3.2	66/3.7	69/*5.5*
Subject 10	72	94/6/88	97/5/92	77/4/73	109/4/105
	NA/2.7	NA/3.0	NA/3.0	NA/4.1	NA/*5.6*
Subject 11	130	133/2/131	124/5/119	108/3/105	128/3/125
	74/3.3	76/3.6	70/3.0	78/4.3	80/*5.4*
Subject 12	107	125/*26*/99	118/*21*/97	90/19/71	114/13/101
	71/3.4	71/3.2	72/3.1	71/3.2	72/*4.0*

*MAP* mean arterial pressure, *ICP* intracranial pressure, *CPP* cerebral perfusion pressure, calculated as per MAP–ICP. T0 shows only values of MAP as ICP probe was inserted after injury (T1). Patients with high ICP indicated in bold, ^a^ = CPP below 50 mmHg requiring metaraminol infusion. Venous saturation of oxygen (Sv02) expressed in %. Continuous cardiac output (CCO) expressed in liters per minute (L/min). Bold values indicate ICP higher then normal. Italic values indicate the increase in Cardiac Output after transfusion

**Table 4:** Hemoglobin levels (g/dL) for all subjects over time

Subjects	Times				
	T0	T1	T2	T3	T4
Sheep 1	10.6	10.0	7.5	7.6	**9.1**
Sheep 2	8.0	8.2	6.4	6.4	**7.6**
Sheep 3	7.0	7.0	5.6	5.1	**6.9**
Sheep 4	8.4	8.2	6.8	6.7	**7.9**
Sheep 5	7.7	7.5	5.2	5.5	**7.0**
Sheep 6	8.6	8.5	6.7	6.4	**8.0**
Sheep 7	9.2	9.0	6.6	6.5	**8.0**
Sheep 8	8.5	8.3	6.5	6.3	**7.9**
Sheep 9	9.8	9.0	6.6	6.5	7.0
Sheep 10	8.7	8.5	6.6	6.7	7.7
Sheep 11	9.0	8.9	6.6	6.9	7.9
Sheep 12	8.2	8.1	6.4	6.2	7.0

Hemoglobin levels at baseline (T0) and after trauma (T1) were reduced by 30% on each subject and maintained during the anaemia phase (T2 and T3) followed by self-transfusion (T4). Hemoglobin for the subjects that experienced an increase in their Hbl after transfusion are indicated in bold

**Table 5:** Partial pressure of tissue oxygenation (PtiO_2_) expressed in mmHg for all subjects over time

PtiO_2_ (mmHg)	T0	T1	T2	T3	T4
Sheep 1	NA	6.3	*2.8*	*2.5*	**11.4**
Sheep 2	NA	0.8	*1.6*	*1.0*	1.67
Sheep 3	NA	2.7	*1.6*	*0.7*	**10.2**
Sheep 4	NA	14.6	17.9	24.1	**34.4**
Sheep 5	NA	0.1	*1.4*	*1.9*	**10.6**
Sheep 6	NA	3.2	*3.1*	*2.7*	**10.6**
Sheep 7	NA	20	35	23	17
Sheep 8	NA	4.2	*6.4*	*15*	**34.3**
Sheep 9	NA	15.5	26.6	28.9	**34.8**
Sheep 10	NA	14.4	*6.4*	*9.7*	**22.9**
Sheep 11	NA	42	31.4	27.5	**37.7**
Sheep 12	NA	49.9	34.5	40	**63**

T0 represents pre-injury phase in which craniectomies are not formalized. PtiO_2_ probes were inserted after trauma, recording PtiO_2_values only from T1

T2 and T3 italic values represent PtiO_2_values below the theoretical critical ischemic threshold

PtiO_2_ at T4 bold values were significantly increased when compared with pre-transfusion times

### RMBF analysis

**Fig. 2:**
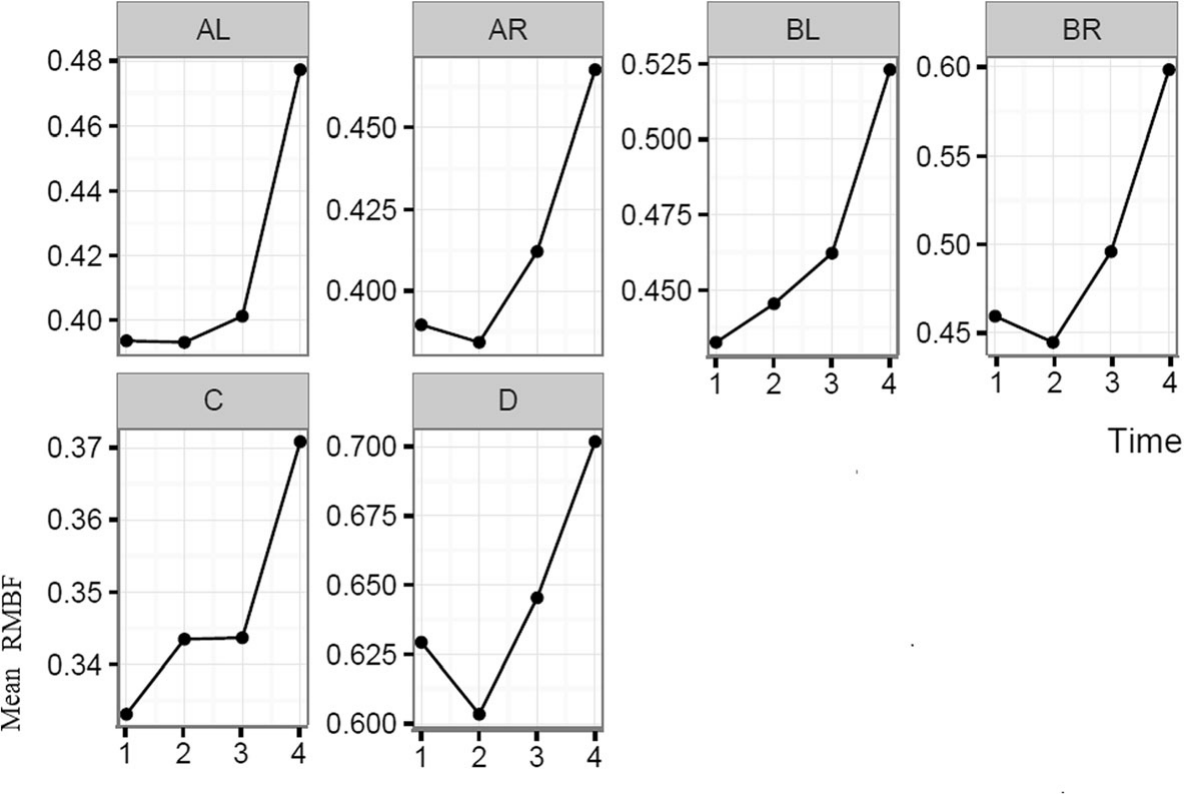
Mean regional microcirculation blood flow (RMBF) from baseline for all subjects at each anatomical region of interest, over time. Time-points (T1–T4) on the *x* axis; mean RMBF on the *y* axis

**Fig. 3:**
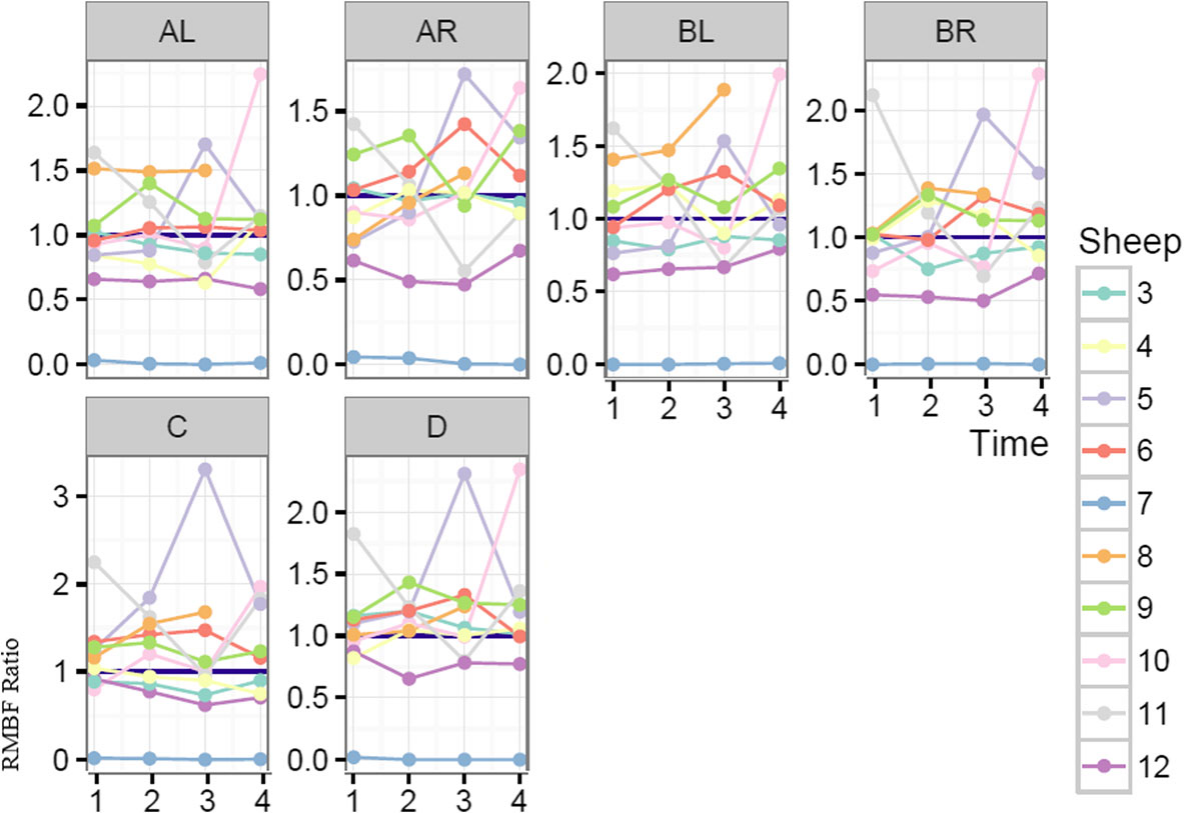
Regional microcirculation blood flow (RMBF) ratio from baseline (*y* axis), per subject and anatomical region of interest, over time (*x* axis)

**Fig. 4:**
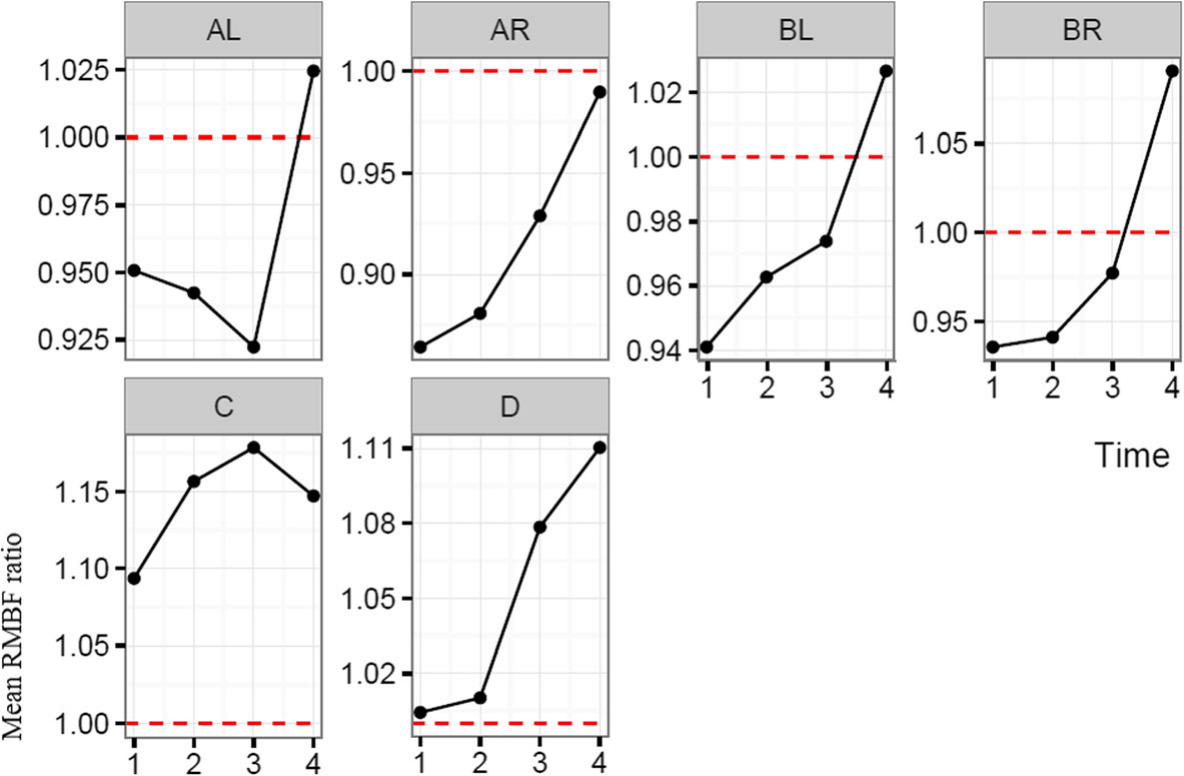
Mean regional microcirculation blood flow (RMBF) ratio from baseline (*y* axis), for all subjects at each anatomical region of interest, over time (*x* axis)

**Fig. 5:**
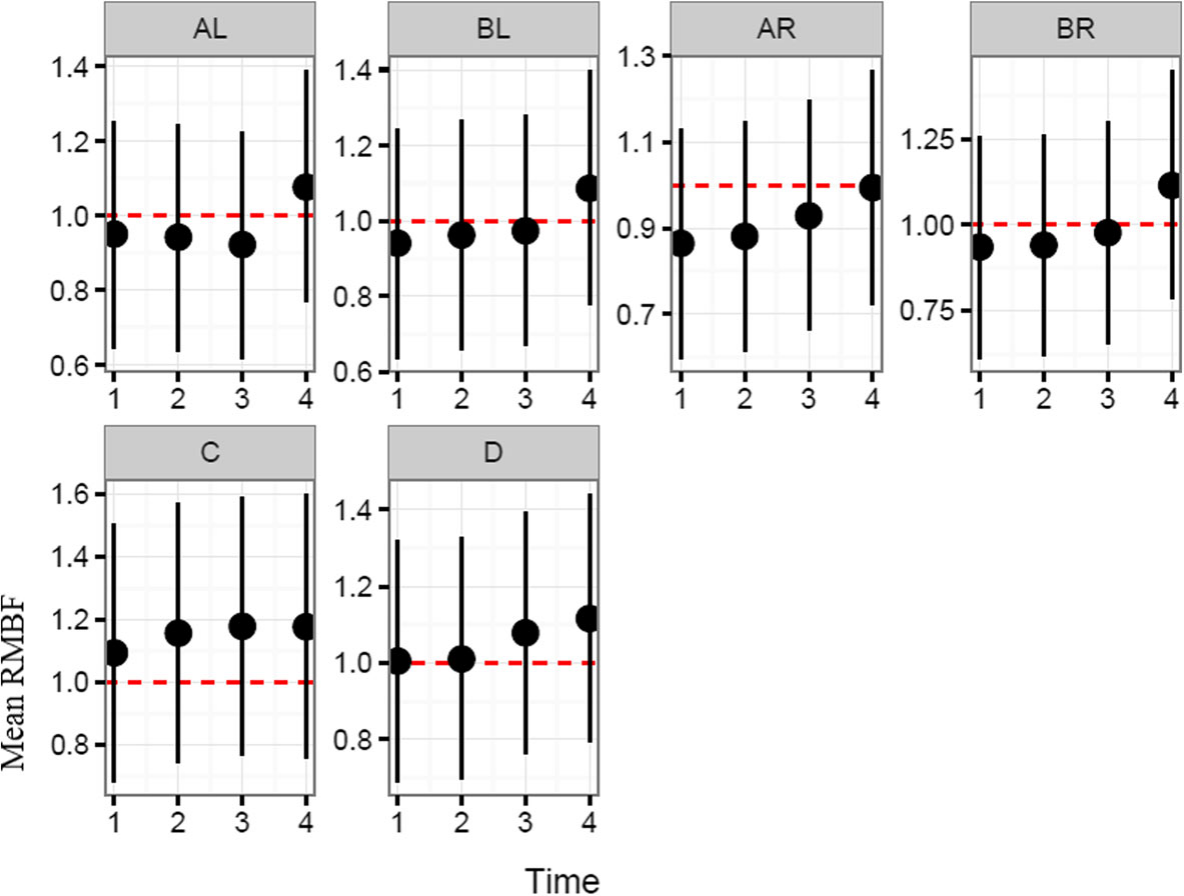
Mean regional microcirculation blood flow (RMBF) ratio from baseline (*y* axis) and standard deviation, for all subjects at each anatomical region of interest over time (*x* axis)

Each sheep had a baseline RMBF measure prior to injury (time zero–T0) and subsequent hourly RMBF measures every hour for 4 h. RMBF values for each subject and tissue region over the entire study time are represented in Fig. 1 with their means represented in Fig. 2; showing that in all subjects, RMBF from baseline to T3 was reduced to ischemic thresholds (20–30 ml/100 g/min) with a considerable increase beyond normal RMBF ranges after transfusion. The ratio of RMBF from T1 (post injury) to T2–T3 (post anaemia) and T4 (post-transfusion) compared to baseline (time zero–T0) was also plotted. A ratio of one indicates absence of changes in RMBF throughout interventions when compared with baseline; RMBF values below the ratio of one represents a reduction in RMBF over time from baseline; RMBF values above such ratio represents an increase in RMBF over time from baseline Fig. 3. RMBF mean ratios from baseline of all subjects, anatomical region, and time are represented in Fig. 4. These two previous figures show that while most subjects were distributed along the ratio of one over all time-points; all their RMBF means at AL, BL, AR, and BR were reduced from baseline to post-injury and anemia phase (T1 to T3) but increased from baseline during transfusion phase (T4). Conversely, the anatomical regions showing a mean RMBF increase from baseline were the ipsilateral thalamus (C) and medulla (D). RMBF means per region and time with the 95% confidence intervals represented by the vertical lines are shown in Fig. 5; statistical significance is represented by those confidence intervals that do not cross the horizontal reference line of 1.

### APP scoring

Results for APP scoring are represented in Table 6. Mild APP staining pattern was seen predominantly in all anatomical regions with moderate and severe APP staining pattern also present and distributed homogeneously over all anatomical regions, except for ipsilateral thalamus showing a predominant “moderate” qualitative score for axonal injury.

**Table 6:** Amyloid precursor protein (APP) staining qualitative scores by tissue region after a severe head injury model

Anatomical regions	Number of subjects categorised on each qualitative APP score		
	Mild	Moderate	Severe
AL	11	1	0
AR	9	2	1
BL	8	3	1
BR	8	3	1
C	4	7	1
D	10	2	0

APP staining categories for each anatomical region amongst six of the eight subjects

## Discussion

The most important finding in this study is the consistent reduction of the mean RMBF from baseline (RMBF pre-injury or T0) and after injury, in all subjects. In addition, the RMBF nadir was found to be located at the core of the contusion, and ongoing RMBF reductions were identified at the ischemic penumbra and at contralateral sites to the contusion (Fig. 4). This is consistent with the a priori expectations regarding cerebral microcirculation after severe head injury although this reduction in RMBF was not seen to be statistically significant (Fig. 5). It is likely that the lack of statistical significance relates to the limited cohort of subjects used in this study; however, although statistical significance adds methodological robustness, experimental studies focus their relevance in their design and plausibility of findings, both of which are demonstrated in this study. These findings contrast with the increase in RMBF from baseline, at the ipsilateral thalamus (C region) and medulla (D region). A possible explanation to the sustained increase in RMBF at these two anatomical regions may be related to the physiological distribution of cerebral blood flow and microcirculation in these areas. Blood supply at the thalamus, derives from multiple arteries (posterior communicating artery, the posterior cerebral artery, the choroidal arteries and in some individuals the Percheron artery). Likewise, blood supply to the medulla, also derives from multiple arteries (the anterior spinal artery, the posterior spinal artery, and direct branches of the vertebral artery). These anatomical peculiarities may be a contributing factor to ensuring blood supply to these two pivotal and confluent anatomical regions of the brain.

The second most relevant finding in this study is the increase of the mean RMBF at all anatomical regions of interest, after the transfusion phase (T4). It may be that the relative increase in subject’s CO could have led to an improvement in microcirculation and subsequent RMBF. However, these subjects’ CPP did not increase as a response to self-transfusion, implying that there was either a compensation through changes on vascular resistances or a variation in cerebral hemodynamics beyond the limits of perfusion pressure.

The third relevant finding was that when RMBF was analysed by ICP groups, those subjects with high ICP (SN 1, SN 2, SN 4, SN 5, and SN 7) had persistently lower RMBF levels than subjects with normal ICP. Furthermore, amongst subjects with high ICP, RMBF was below the ischemic threshold at all-time points and throughout several anatomical regions. Such finding is consistent with patho-physiologic expectations in the context of severe head injury, increasing the internal validity and reliability of our findings.

**Fig. 6:**
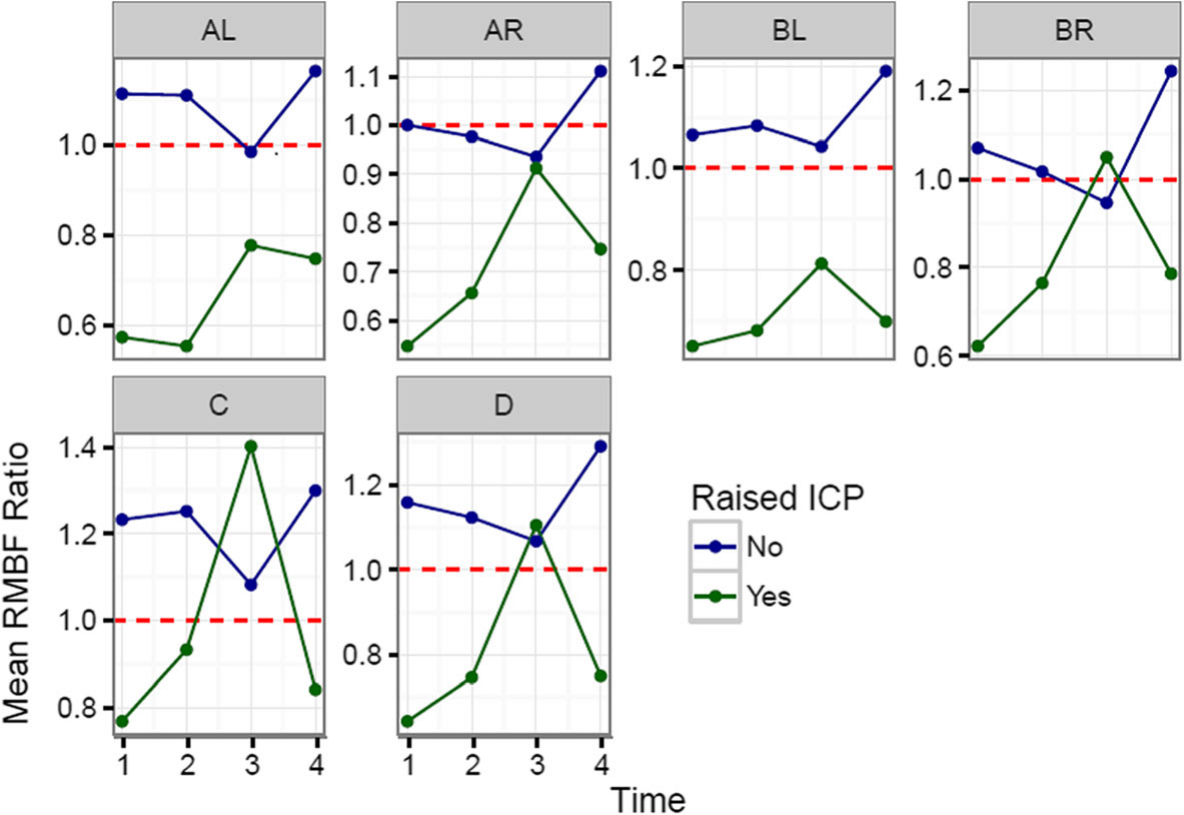
Mean regional microcirculation blood flow (RMBF) ratio from baseline (*y* axis), for all subjects at each anatomical region of interest, over time (*x* axis), comparing two cohorts of subjects (high ICP versus normal ICP)

To elucidate the impact of high ICP on RMBF over time after injury, we analysed the mean RMBF ratio from baseline in two separated cohorts: those subjects with normal ICP and those with raised ICP (Fig. 6) and found that

- Subjects with normal ICP values (defined by an ICP < 20 mmHg) had an increase in their mean RMBF from baseline after trauma (their mean RMBF was above the ratio of 1) as opposed to subjects with raised ICP, who had a reduced mean RMBF from baseline after trauma (their mean RMBF was below the ratio of 1).
- Subjects with normal ICP showed their lowest mean RMBF at T3 (just prior to transfusion and after 2 h from trauma) and an increase in their mean RMBF at T4 (after self-transfusion) as opposed to subjects with raised ICP.

The time-dependent reduction of cerebral RMBF in all regions from baseline, amongst subjects with raised ICP is a relevant finding as it may indicate that even in situations where severe tissue disruption is not derived from the primary injury leading to a mild degree of APP staining, there is still the potential for the development of cerebral infarcts.

The fourth relevant finding was that PtiO_2_ values declined during the anemic phase and had a statistically significant increase after autologous transfusion, in all subjects except for SN 2 and 7, a finding consistent with previous studies [21]. It may be that despite the global reduction in RMBF from baseline, the maintenance of RMBF above ischemic-thresholds may be sufficient to preserve tissue oxygenation; this emphasizes the need to preserve cerebral perfusion even from early hours after trauma.

**Fig. 7:**
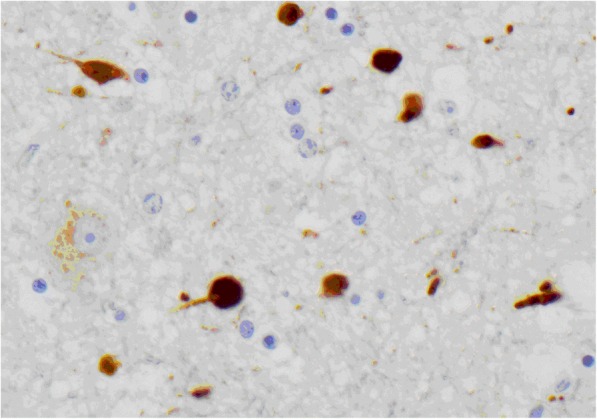
Axonal retraction balls (ARB) in a subject with severe head injury and diffuse axonal injury

In addition, histopathological analysis using APP staining (Table 6) showed a mild degree of APP staining not expected from the severity of the injury. However, moderate and severe APP staining scores were also found along all regions including the contralateral hemisphere, demonstrating that axonal injury is globally manifested. Indeed, the presence of axonal retraction balls (ARB) corroborates with the severity of injury, as ARB are considered the hallmark of DAI (Fig. 7). This study compares for the first time the interrelation between tissue damage and microcirculation in experimental models at specific anatomical regions. It is the first time that cerebral histopathology and quantification of microcirculation post-trauma are analysed simultaneously; showing how cerebral blood flow and axonal tissue are temporal and spatially affected after trauma.

These observations are clinically relevant, they demonstrate the increasing vulnerability of cerebral microcirculation not just in relation to the severity of the injury but most importantly to the superposition of additional insults such as anemia and increased ICP. In addition, the significant increase in PtiO_2_ after transfusion reflects a degree of reversibility and brings the question onto its theoretical role in reducing the risk of cerebral infarcts and improving neurological or functional outcomes.

In summary, in the presence of severe head injury and superimposed anemia, cerebral microcirculation is consistently reduced from baseline with a nadir at the level of the core of the contusion and a maximal reduction at the pre-transfusion phase. In such scenario, when raised ICP is added, cerebral microcirculation is reduced further and does not recover with autologous transfusion. Moreover, during anemia, PtiO_2_ values are reduced from baseline with a statistically significant increase after autologous transfusion.

### Study limitations

The biggest limitation of this study relies on the limited capacity to clinically extrapolate conclusions raised from experimental models. However, it is from experimental studies that direct quantification of cerebral microcirculation becomes feasible. Another important limitation relates to the limited length of the time during which the animals were monitored and studied. Hence, although longer monitoring time could have demonstrated a wider range of patho-physiological processes affecting microcirculation, the focus of this study was to capture the early changes in RMBF at specific regions of interest superimposed to the early histopathology changes derived from axonal injury after trauma.

## Conclusion

Cerebral microcirculation after severe head injury is consistently reduced from baseline, at the ipsilateral and contralateral site of the contusion. When normovolemic anemia is superimposed, cerebral microcirculation reaches its nadir with an increase response after autologous transfusion amongst subjects with normal ICP, unlike for subjects with high intracranial pressures. The reduction in cerebral microcirculation is associated with the severe APP staining and the presence of axonal retraction balls, the DAI histological hallmark. Cerebral oxymetry showed a statistically significant increase from ischemic to above-normal values after autologous transfusion.

## Additional file

Additional file 1: Metabolic variables. (DOCX 119 kb)

## Abbreviations

APP: Amyloid precursor protein
ARB: Axonal retraction balls
CO: Cardiac output
CPP: Cerebral perfusion pressure
CVP: Central venous pressure
ICE: Intracardiac echocardiography
ICP: Intracranial pressure
IMT: Interactive Medical Technology
PaC02: Partial pressure of arterial C02
PaO_2_: Partial pressure of oxygen
PtiO_2_: Partial pressure of tissue oxygenation
RMBF: Regional microcirculation blood flow
SVc02: Central venous oxygen saturation
