# Supplementary material for: The effects of normovolemic anemia and blood transfusion on cerebral microcirculation after severe head injury
Source: Intensive Care Med Exp. 2018 Nov 8;6:46. doi: 10.1186/s40635-018-0210-5 (PMC6223395; doi:10.1186/s40635-018-0210-5)
Supplement: Supplementary file 1 — Metabolic variables. (DOCX 119 kb) [file 40635_2018_210_MOESM1_ESM.docx]

**Additional file 1**

**Metabolic variables**

Metabolic variables directly affecting oxygen delivery to tissues and cerebral blood volume, such as Pa02 and partial pressure of arterial C02 (PaC02) respectively, remained stable throughout the study with minimal variations amongst subjects, as shown below in the table.

PH / PC02 and P02 *(at Fi02 0.4)* values per sheep at each time points:

| **Subjects** | **Times** | | | | |
| --- | --- | --- | --- | --- | --- |
|  | **T0**  PH /PC02 /P02 (mmHg) | **T1**  PH /PC02 /P02 (mmHg) | **T2**  PH /PC02 /P02 (mmHg) | **T3**  PH /PC02 /P02 (mmHg) | **T4**  PH /PC02 /P02 (mmHg) |
| **Sheep 1** | 7.45/37/204 | 7.43/38.6/186 | 7.39/43/175 | 7.38/37/176 | 7.41/42/181 |
| **Sheep 2** | 7.36/43/176 | 7.46/38/217 | 7.47/34/218 | 7.48/33/217 | 7.49/33.1/220 |
| **Sheep 3** | 7.40/40/208 | 7.40/38.7/212 | 7.41/38.1/209 | 7.39/38.9/200 | 7.42/36.7/209 |
| **Sheep 4** | 7.41/47.1/213 | 7.45/40.8/221 | 7.45/39.8/216 | 7.44/39.1/220 | 7.47/39.2/219 |
| **Sheep 5** | 7.35/49.1/173 | 7.35/48.0/198 | 7.37/45.3/196 | 7.39/44.0/194 | 7.40/43.7/191 |
| **Sheep 6** | 7.43/44.0/196 | 7.41/42.0/182 | 7.41/42.4/190 | 7.38/43.4/196 | 7.39/45.0/199 |
| **Sheep 7** | 7.35/45.9/238 | 7.46/36.6/280 | 7.38/43.9/319 | 7.41/41.4/311 | 7.45/39.2/320 |
| **Sheep 8** | 7.40/41.7/212 | 7.39/44.4/216 | 7.41/45.1/246 | 7.41/45.9/249 | 7.41/49.5/241 |
| **Sheep 9** | 7.36/46.4/192 | 7.35/45.9/191 | 7.35/47.0/190 | 7.39/44.3/175 | 7.35/46.6/183 |
| **Sheep 10** | 7.38/51/205 | 7.38/51/208 | 7.41/47/207 | 7.42/47/208 | 7.40/47/207 |
| **Sheep 11** | 7.36/46/184 | 7.34/47/185 | 7.33/43/195 | 7.31/42/187 | 7.33/44/185 |
| **Sheep 12** | 7.32/46/201 | 7.31/44.6/203 | 7.31/40.9/174 | 7.30/46/201 | 7.31/48/199 |

All subjects were ventilated using an oxygen inspired fraction of (Fi02) of 0.4 (40% oxygen). PaC02 was maintained within normocapnic ranges.

**Extra-cranial tissues cytometric count:**

Direct quantification of RMBF was also performed at extra-cranial regions at the skin, heart, kidney and spleen. The aim was to demonstrate systemic distribution of color coded microspheres as a proof of concept during all time points. In addition, trace levels of RMBF at the spleen aimed to demonstrate that spleen artery ligation had been performed efficiently.

Mean Regional Microcirculation Blood Flow (RMBF) at extracranial tissues, over time:


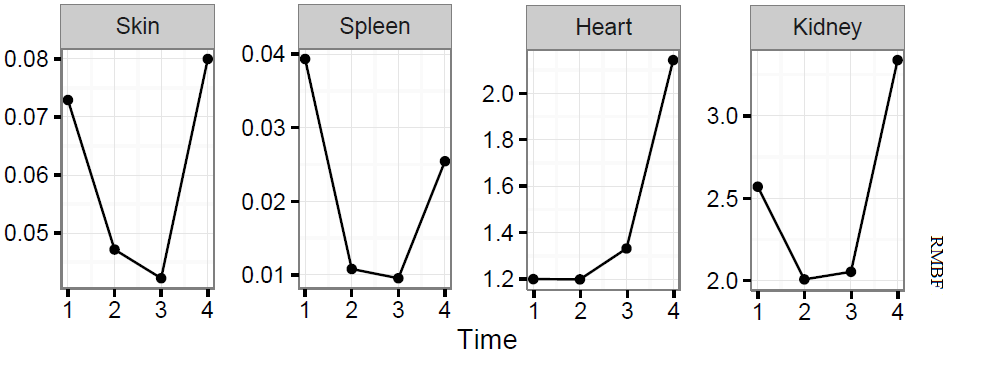


RMBF in spleen showed negligible perfusion as expected from the arterial spleen ligation completed in the pre-injury phase. This finding confirmed the efficiency of the splenic artery ligation as proven by the necrotic aspect of the spleen after harvesting and corroborated by the stable hemoglobin levels throughout the study suggesting that changes on cerebral RMBF and PtiO_2_ prior to the anemia phase, were not related to changes in hemoglobin.

**Statistical comparison with a previous study**

A statistical comparison of the RMBF from T2 to T4 between a severe head injury model without anemia or transfusion and the current study (severe head injury model with anemia and self-transfusion) was completed and shown in figure below.


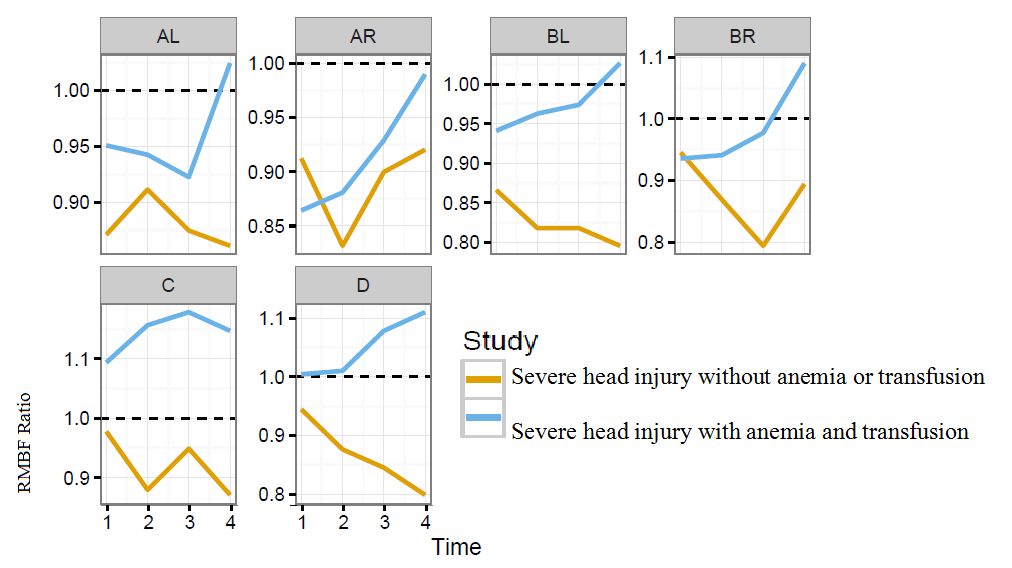


No statistical significance was found between their RMBF distribution and quantification at any of the anatomical regions of interest or time points.

However, mean RMBF within the anemia and self-transfusion cohort increased from baseline after transfusion and at all time-points after injury at thalamus and medulla. Although it appears to be paradoxical, it raises the possibility that the maintenance of normovolemia and the superimposed transfusion, could have led to a certain degree of hyperaemia within cerebral microcirculation, leading to such a paradoxical increase in RMBF.

Comparison of mean RMBF from T2 to T4 times between two studies (severe head injury without anemia or transfusion and severe head injury with anemia and with transfusion) at each specific anatomical site of interest:

| Tissue | Mean | Lower | Upper |
| --- | --- | --- | --- |
| AL | 0.01 | - 0.06 | 0.08 |
| AR | 0.00 | - 0.06 | 0.06 |
| BL | 0.03 | - 0.04 | 0.10 |
| BR | 0.04 | - 0.05 | 0.13 |
| C | 0.02 | -0.03 | 0.07 |
| D | 0.02 | - 0.08 | 0.13 |

Means and 95% confidence intervals; Confidence intervals that do not include zero, indicate a statistically significant increase in RMBF between both studies.
